# A prediction model for secondary invasive fungal infection among severe SARS-CoV-2 positive patients in ICU

**DOI:** 10.3389/fcimb.2024.1382720

**Published:** 2024-07-08

**Authors:** Leilei Su, Tong Yu, Chunmei Zhang, Pengfei Huo, Zhongyan Zhao

**Affiliations:** ^1^ Department of Critical Care Medicine, China-Japan Union Hospital of Jilin University, Changchun, China; ^2^ Department of Epidemiology, University of Pittsburgh School of Public Health, Pittsburgh, PA, United States

**Keywords:** COVID-19, invasive fungal infection, BMI, intensive care unit, cumulative incidence, logistic model, prediction

## Abstract

**Background:**

The global COVID-19 pandemic has resulted in over seven million deaths, and IFI can further complicate the clinical course of COVID-19. Coinfection of COVID-19 and IFI (secondary IFI) pose significant threats not only to healthcare systems but also to patient lives. After the control measures for COVID-19 were lifted in China, we observed a substantial number of ICU patients developing COVID-19-associated IFI. This creates an urgent need for predictive assessment of COVID-19 patients in the ICU environment for early detection of suspected fungal infection cases.

**Methods:**

This study is a single-center, retrospective research endeavor. We conducted a case-control study on severe acute respiratory syndrome coronavirus 2 (SARS-CoV-2) positive patients. The cases consisted of patients who developed any secondary IFI during their ICU stay at Jilin University China-Japan Union Hospital in Changchun, Jilin Province, China, from December 1st, 2022, to August 31st, 2023. The control group consisted of SARS-CoV-2 positive patients without secondary IFI. Descriptive and comparative analyses were performed, and a logistic regression prediction model for secondary IFI in COVID-19 patients was established. Additionally, we observed an increased incidence of COVID-19-associated pulmonary aspergillosis (CAPA) during this pandemic. Therefore, we conducted a univariate subgroup analysis on top of IFI, using non-CAPA patients as the control subgroup.

**Results:**

From multivariate analysis, the prediction model identified 6 factors that are significantly associated with IFI, including the use of broad-spectrum antibiotics for more than 2 weeks (aOR=4.14, 95% CI 2.03-8.67), fever (aOR=2.3, 95%CI 1.16-4.55), elevated log ^IL-6^ levels (aOR=1.22, 95% CI 1.04-1.43) and prone position ventilation (aOR=2.38, 95%CI 1.15-4.97) as independent risk factors for COVID-19 secondary IFI. High BMI (BMI ≥ 28 kg/m^2^) (aOR=0.85, 95% CI 0.75-0.94) and the use of COVID-19 immunoglobulin (aOR=0.45, 95% CI 0.2-0.97) were identified as independent protective factors against COVID-19 secondary IFI. The Receiver Operating Curve (ROC) area under the curve (AUC) of this model was 0.81, indicating good classification.

**Conclusion:**

We recommend paying special attention for the occurrence of secondary IFI in COVID-19 patients with low BMI (BMI < 28 kg/m^2^), elevated log ^IL-6^ levels and fever. Additionally, during the treatment of COVID-19 patients, we emphasize the importance of minimizing the duration of broad-spectrum antibiotic use and highlight the potential of immunoglobulin application in reducing the incidence of IFI.

## 1 Introduction

Coronavirus Disease 2019 (COVID-19) is a highly contagious disease caused by severe acute respiratory syndrome coronavirus 2 (SARS-CoV-2). COVID-19 pneumonia frequently results in severe respiratory failure during the ongoing global pandemic. Since late 2019, COVID-19 pneumonia, which is often associated with severe respiratory failure during the global pandemic, has resulted in a staggering 767 million confirmed cases and approximately 7 million cumulative deaths globally as of July 2023, according to the World Health Organization ((W.H.O.), 2023). It is worth noting that extensive lung damage caused by SARS-CoV-2 infection has been identified as the leading cause of death (Liu et al., 2022).

However, in the early stages of COVID-19 pneumonia, the impact of secondary invasive bacterial or fungal infections on mortality may have been underestimated, and there are limited reports on patients with COVID-19 pneumonia complicated with fungal infections. Similar to patients with Middle East respiratory syndrome (MERS) and Severe acute respiratory syndrome (SARS), a cytokine storm caused by inflammatory dysregulation may be associated with worse clinical outcomes in COVID-19 patients (Mangalmurti and Hunter, 2020). Both SARS-CoV and SARS-CoV-2 indeed share similar epidemiological, biological, and clinical features (Peeri et al., 2020). Data from the coronavirus outbreak caused by SARS-CoV in 2003 showed that the incidence of fungal infection in critically ill patients was as high as 21.9% to 33%, making it the primary cause of death in SARS patients and accounting for 25% to 73.7% of all deaths (Li and Pan, 2003; Zheng et al., 2003). Therefore, the issue of fungal infection should be treated with utmost seriousness.

Initially, at the onset of SARS-CoV-2 infection confirmation, the likelihood of bacterial and fungal co-infection was low. However, as ICU hospitalization and the need for invasive mechanical ventilation in critically ill patients increased, bacterial and fungal co-infection became common, with the risk escalating alongside the severity of the disease (Rawson et al., 2020). Nevertheless, the incidence of invasive fungal infections (IFI) reported in previous literature varies greatly, primarily due to the heterogeneity of the study populations, surveillance protocols, and diagnostic criteria for fungal infections. Numerous studies have identified risk factors associated with these secondary fungal infections, which include, but are not limited to, the use of corticosteroids or immunosuppressants (such as dexamethasone or tocilizumab), damage to the epithelial barrier, prolonged use of broad-spectrum antibiotics, invasive medical procedures, and extended hospital stays (Cevik et al., 2020; Diao et al., 2020; Ripa et al., 2021).

However, it is worth noting that existing research has primarily focused on secondary IFI in COVID-19 patients compared to non-COVID-19 patients. In the early stages of the COVID-19 pandemic, strict lockdown measures were implemented in China, and these were lifted in December 2022. Subsequently, a significant proportion of hospitalized patients were diagnosed as SARS-CoV-2 positive. During this phase, there has been limited research on secondary IFI in severe COVID-19 patients in the ICU environment. In this study, our aim is to establish a logistic regression prediction model for early detection of suspected cases of secondary IFI in severe COVID-19 patients in the ICU.

## 2 Methods

### 2.1 Study design and population

We conducted a retrospective case-control study, including all adult patients (>18 years old) admitted to the Intensive Care Unit (ICU) of China-Japan Union Hospital of Jilin University, located in Changchun, Jilin Province, China, between December 1st, 2022, and August 31st, 2023, with a positive real-time reverse transcription polymerase chain reaction (RT-PCR) test for SARS-CoV-2. The exclusion criteria included the following: (1) ICU stay fewer than 48 hours, (2) patients’ SARS-CoV-2 PCR was negative upon admission but turned positive during hospitalization, and (3) incomplete clinical data.

The COVID-19 cases included in this study were diagnosed based on the diagnosis and treatment plan for novel coronavirus infection (10th edition, trial) released by the National Health Commission of the People’s Republic of China (China, 2023). We adopted the definition of IFI following the guidelines provided by the European Confederation of Medical Mycology (ECMM) and the International Society for Human and Animal Mycology (ISHAM) (Koehler et al., 2021). Additionally, within the subgroup of patients definitively diagnosed with IFI, we specifically selected those with invasive aspergillosis as the study group, with the control group comprising patients without invasive aspergillosis among IFI cases.

### 2.2 Sample procedures

In this study, respiratory samples and serum used for IFI testing were collected. Upper respiratory tract samples involved obtaining tracheal aspirates (TA) from patients, which were then placed in a sterile, airtight container. Within 1 hour, these samples were delivered to the microbiology laboratory. After confirming them as qualified respiratory specimens, they underwent microscopic examination and culture. The entire process strictly followed the microbiological culture standards of Jilin University China-Japan Union Hospital. Additionally, we collected serum samples for (1,3)-β-D-glucan(BDG) testing, Galactomannan(GM) testing, and respiratory virus multiplex PCR testing. If (1) the serum BDG or GM test showed a positive result, and (2) within 3 days, the upper respiratory tract pathogen test did not detect any pathogens, and (3) the respiratory virus multiplex PCR test was negative, and (4) the patient required mechanical ventilation due to respiratory failure, we then chose to obtain bronchoalveolar lavage fluid or tracheal aspirates for the second-generation metagenomic sequencing (mNGS) (Diseases, 2020) test using fiberoptic bronchoscopy. The mNGS is a diagnostic technique for identifying pathogens in pulmonary infections. It exhibits greater sensitivity, particularly for anaerobic bacteria, fungi, Pneumocystis, etc., compared to microbial culture (50.7% vs. 35.2%). This method allows for the rapid and comprehensive detection of nucleic acids from a wide range of pathogens (Brown et al., 2018; Simner et al., 2018). Employing high-throughput sequencing technology, it analyzes microbial nucleic acid sequences in samples, identifies microorganisms by comparing them with existing sequences in the database, and holds significant clinical value for clinically suspected, challenging, or immunocompromised patients. However, due to the relatively high cost of mNGS, samples (tracheal aspirate or bronchoalveolar lavage - BAL) are collected for mNGS testing only with the consent of the patient or their family. This is done to assist in the rational selection of antibiotics. The testing reports are provided by the Tianjin Golden Key Medical Laboratory.

### 2.3 Case definition

According to the ECMM/ISHAM guidelines, the diagnosis of all IFI in this study relies primarily on clinical/radiological and mycological evidence. Patients diagnosed with IFI must present all three of the following pieces of evidence: (1) Clinical Features: Intermittent fever lasting for more than 3 days; Onset of new fever for more than 48 hours after defervescence during appropriate antifungal therapy; Deterioration of respiratory status; Hemoptysis, pleuritic friction, or chest pain. (2) Radiological Evidence: Chest CT showing pulmonary infiltrates, nodules, or cavities. (3) Mycological Evidence: Direct cultivation of fungal pathogens from respiratory samples (TA or BAL).

Among IFI cases, we defined COVID-19-associated pulmonary aspergillosis (CAPA) as meeting the following criteria: (1) Radiological Criteria: Chest CT showing pulmonary infiltrates, nodules, or cavities. (2) Mycological Evidence: Fungal mycological evidence obtained from BAL or TA, including cultivation or mNGS. Serum GM test result ≥ 0.25.

We performed the IFI testing (upper respiratory and serum samples) for all COVID-19 patients. To ensure accurate classification, each COVID-19 patient meeting the aforementioned criteria underwent consultation involving one microbiologist and two critical care medicine specialists. Implementing this rigorous approach, we aimed to uphold the integrity of the study and ensure the accuracy of classification.

### 2.4 Treatment principles

Within 24 hours of all patients being admitted to the ICU, we conducted necessary examinations, including complete blood count, Interleukin-6 (IL-6), C-reactive protein (CRP), arterial blood gas analysis, serum galactomannan, serum (1,3)-β-D-glucan, chest imaging, and targeted examinations for patients with underlying conditions.

#### 2.4.1 Antiviral treatment

##### 2.4.1.1 Nirmatrelvir/ritonavir

For patients with mild to moderate symptoms progressing to severe within 5 days, we use a combination of remdesivir and lopinavir/ritonavir. Remdesivir 300mg and lopinavir 100mg are taken together every 12 hours for 5 days.

##### 2.4.1.2 Azvudine

Widely used for patients with moderate COVID-19 infection. A dosage of 5mg per day for a maximum of 14 days.

##### 2.4.1.3 Monolaurin

Applied to patients with mild to moderate symptoms progressing to severe within 5 days. A dose of 800mg is taken orally every 12 hours for 5 days.

##### 2.4.1.4 COVID-19 Immunoglobulin

Administered early in the course to patients with severe risk factors, high viral load, and rapid disease progression. Dosage is medium: 200mg/kg, severe and critical: 400mg/kg. Repeat infusions may occur based on patient improvement, not exceeding a total of 5 times.

#### 2.4.2 Immunotherapy

##### 2.4.2.1 Glucocorticoids

Short-term use (not exceeding 10 days) for severe and critical cases with worsening oxygenation, rapid radiological progression, and excessive activation of systemic inflammatory response. Dexamethasone 5mg/day or methylprednisolone 40mg/day.

##### 2.4.2.2 Tocilizumab

Administered to severe and critical cases with significantly elevated IL-6 levels.

#### 2.4.3 Anticoagulant therapy

Low molecular weight heparin is administered to cases with severe risk factors and rapid disease progression, without contraindications.

#### 2.4.4 Antibiotic treatment

Empirical use of broad-spectrum antibiotics for patients with elevated white blood cell and neutrophil counts on admission, combined with chest imaging suggestive of community-acquired pneumonia or secondary bacterial infection.

#### 2.4.5 Respiratory support

Implementation of standardized and effective oxygen therapy measures based on the patient’s condition, such as high-flow nasal oxygen or non-invasive ventilation. In cases with PaO2/FiO2 below 200mmHg and significant respiratory distress, invasive mechanical ventilation is initiated, supplemented with prone positioning for at least 12 hours daily.

#### 2.4.6 Circulatory support

For critically ill cases with shock, after adequate fluid resuscitation, central venous access is established, and vasoactive drugs are employed.

#### 2.4.7 Continuous renal replacement therapy

Considered for critically ill cases with acute kidney injury, particularly those with hyperkalemia, severe acidosis, ineffective diuretics, pulmonary edema, or excessive fluid overload.

All treatment plans conducted in the ICU, including invasive procedures, have been pre-approved by patients or their families. This study was approved by the Ethics Committee of China-Japan Union Hospital of Jilin University, with the approval number 2023-KYYS-092.

### 2.5 Statistical analysis

Overall cumulative incidence of IFI, overall 28-day mortality, as well as 28-day mortality by IFI and by CAPA in the study population were calculated. Differences of incidence by groups were tested using two-sample t-test for proportions. Characteristics of cases and controls were summarized and compared. We used Shapiro-Wilk test and 0.05 as the threshold to evaluate normality of continuous covariates by cases and controls. Two-sample student’s t tests with unequal variance assumption were used if covariates follow normal distribution in both cases and controls, otherwise, Mann-Whitney U tests were used for comparison of continuous covariates. We used Chi-Squared test to compare distribution of categorical covariates if all cells’ expected count was > 5, otherwise, Fisher’s exact tests were used. To establish a prediction model for IFI, we considered stepwise model selection for model building, and we used forward model selection due to non-convergence in the full model. Potential risk factors except factors with <2 patients in either case or control group were included in the model selection. The following variables were inputted into the selection process: Age, Gender, BMI, COVID-19 severity, Onset to admission days, Surgery history, Long-term glucocorticoid use before admission, Chronic obstructive pulmonary disease (COPD), Chronic bronchitis, Liver disease, Hypertension, Hematologic malignancies, Organ transplantation, Immunosuppressive therapy, APACHE II score, Positive influenza virus nucleic acid, Fever, Type I respiratory failure, Type II respiratory failure, Deterioration in ventilatory parameters, White blood cell count, Neutrophil count, Lymphocyte count, Neutrophil to Lymphocyte ratio (NLR), Log ^CRP^, Glycated hemoglobin, Log ^IL-6^, TNF-alpha, Invasive mechanical ventilation (IMV), IMV*IMV days, the interaction term between IMV and IMV days, Parenteral nutrition, Methyl prednisolone, Dexamethasone, Tocilizumab, Broad-spectrum antibiotics (greater than 2 weeks), Continuous Renal Replacement Therapy(CRRT), Extracorporeal Membrane Oxygenation(ECMO), Deep venipuncture, Bronchoscopy, Prone position ventilation, Tracheotomy, Azivudine, Anticoagulant, COVID-19 immunoglobulin. IMV and IMV days were entered as IMV (yes/no) and the interaction term between IMV and IMV days into model selection. We then used the selected logistic regression models to predict IFI and estimate odds ratios and corresponding 95% confidence intervals (CIs) for each potential risk factor. Model performance was evaluated using area under the curve (AUC) in the Receiver Operating Curve (ROC). A level of 0.05 for type I error rate was used as the cutoff for statistical significance. All analyses were conducted using R version 4.2.1. R Core Team (2022). R: A language and environment for statistical computing. R Foundation for Statistical Computing, Vienna, Austria. URL https://www.R-project.org/.

## 3 Result

**Figure 1:**
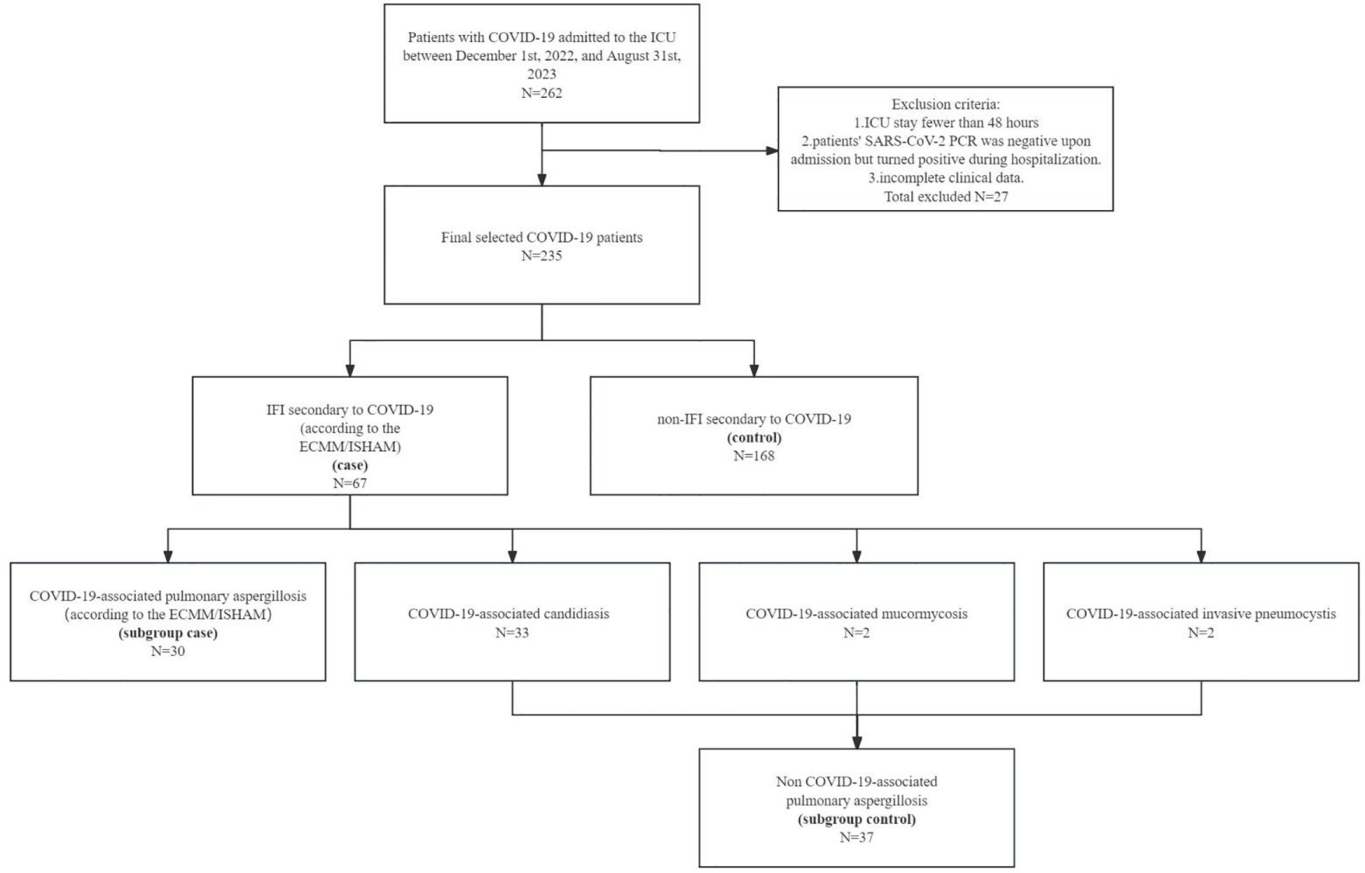
CONSORT diagram of study selection.

From December 1st, 2022, to August 31st, 2023, a total of 262 adult patients with COVID-19 were admitted to the ICU at China-Japan Union Hospital of Jilin University. After excluding 27 inpatients who had a hospitalization duration in the ICU of less than 48 hours, those who contracted the new coronavirus after admission, and individuals with incomplete clinical data, we included 235 hospitalized patients in the final analysis set (**Figure 1**). Among the 235 patients included in the analysis, the cumulative incidence of secondary IFI was 28.5% (67/235), and the 28-day mortality in this ICU study population was 35.3% (83/235). The mortality in IFI was 40.3% (27/67), in non-IFI was 33.3% (56/168). The mortality in CAPA was 33.3% (10/30), in non-CAPA was 46% (17/37). Baseline characteristics, as well as other clinical and laboratory variables of the 235 patients by IFI status, were summarized in **Table 1**. The median age in the study population was 72 years (IQR 62.5-78 years), with 160 out of 235 (68.1%) being male patients. The majority of the 235 patients were categorized as having a normal or overweight BMI according to the Chinese-population specific BMI cutoff (China, 2013), with a median BMI of 23.9 kg/m^2^ (IQR 22.1-25.9 kg/m^2^). According to the diagnosis and treatment plan for novel coronavirus infection (10th edition, trial), 219 out of 235 (93.2%) were classified as having a “severe” or “critical” COVID-infection type.

**Table 1:** Characteristics and related clinical factors by IFI of COVID-19 patients.

Characteristics	Overall	Non-IFI	IFI	P-value
	N=235	N=168	N=67	
Age(years), median (IQR)	72.0[62.5;78.0]	72.0[62.0;78.0]	71.0[66.0;78.0]	0.857
Gender:				0.334
female	75 (31.9%)	50 (29.8%)	25 (37.3%)	
male	160 (68.1%)	118 (70.2%)	42 (62.7%)	
BMI (kg/m^2^), median (IQR)	23.9 [22.1;25.9]	24.1 [22.6;26.4]	23.2 [21.3;25.4]	0.019
BMI categories:				0.360
Normal	109 (46.4%)	74 (44%)	35 (52.2%)	
Underweight	10 (4.3%)	6 (3.6%)	4 (6.0%)	
Overweight	88 (37.4%)	65 (38.7%)	23 (34.3%)	
Obesity	28 (11.9%)	23 (13.7%)	5 (7.5%)	
ICU stay, median (IQR)	10.0 [6.0;13.0]	9.0 [5.0;13.0]	13.0 [9.0;16.5]	<0.001
IMV days, median (IQR)	0.0 [0.0;6.3]	0.0 [0.0;3.7]	4.6 [0.0;10.8]	<0.001
Severity scores				
COVID-19 severity:				0.002
Moderate	16 (6.8%)	15 (8.9%)	1 (1.5%)	
Severe	97 (41.3%)	77 (45.8%)	20 (29.9%)	
Critical	122 (51.9%)	76 (45.2%)	46 (68.7%)	
APACHE II score, median (IQR)	19.0 [14.0;26.0]	18.0 [13.0;25.0]	20.0 [16.5;27.5]	0.076
SOFA score >2	25 (10.6%)	12 (7.1%)	13 (19.4%)	0.012
Persistent (>24h) or Recurrent fever	79 (33.6%)	44 (26.2%)	35 (52.2%)	<0.001
Positive influenza virus nucleic acid	11 (4.7%)	5 (3.0%)	6 (9.0%)	0.081
Worsening of respiratory support	77 (32.8%)	45 (26.8%)	32 (47.8%)	0.003
Basic illness				
Diabetes	96 (40.9%)	67 (39.9%)	29 (43.3%)	0.740
COPD	15 (6.4%)	9 (5.4%)	6 (9.0%)	0.375
Hypertension	127 (54.0%)	91 (54.2%)	36 (53.7%)	1.000
Data before admission to ICU				
Onset to admission, median (IQR)	7.0 [5.0;10.0]	7.0 [5.0;10.0]	7.0 [5.5;11.5]	0.858
Long-term glucocorticoid use before admission	23 (9.8%)	12 (7.1%)	11 (16.4%)	0.055
Treatment and intervention				
Broad-spectrum antibiotics greater than 2 weeks	87 (37.0%)	45 (26.8%)	42 (62.7%)	<0.001
Vascular active drug	89 (37.9%)	55 (32.7%)	34 (50.7%)	0.016
IMV	94 (40.0%)	55 (32.7%)	39 (58.2%)	0.001
Prone-position ventilation	73 (31.1%)	39 (23.2%)	34 (50.7%)	<0.001
Deep venipuncture	91 (38.7%)	56 (33.3%)	35 (52.2%)	0.011
Bronchoscopy	43 (18.3%)	20 (11.9%)	23 (34.3%)	<0.001
CRRT	42 (17.9%)	28 (16.7%)	14 (20.9%)	0.565
Nematrevir/ritonavir	98 (41.7%)	67 (39.9%)	31 (46.3%)	0.453
Azivudine	65 (27.7%)	54 (32.1%)	11 (16.4%)	0.023
Monolaurin	14 (6.0%)	11 (6.5%)	3 (4.5%)	0.762
Anticoagulant therapy	194 (82.6%)	138 (82.1%)	56 (83.6%)	0.943
COVID-19 immunoglobulin	67 (28.5%)	53 (31.5%)	14 (20.9%)	0.141
Methyl prednisolone	153 (65.1%)	113 (67.3%)	40 (59.7%)	0.344
Dexamethasone	41 (17.4%)	30 (17.9%)	11 (16.4%)	0.943
Tocilizumab	13 (5.5%)	10 (6.0%)	3 (4.5%)	0.763
Laboratory data				
Serum GM positive	32 (13.6%)	11 (6.5%)	21 (31.3%)	<0.001
Serum BDG positive	73 (31.1%)	42 (25.0%)	31 (46.3%)	0.002
Log ^IL-6^, median (IQR)	2.5 [1.1;4.0]	2.2 [0.9;3.7]	3.2 [1.7;4.6]	0.003
White blood cell count, median (IQR)	8.4 [5.5;11.9]	7.6 [5.2;11.4]	9.8 [6.4;12.9]	0.021
Neutrophil count, median (IQR)	7.0 [4.6;10.7]	6.3 [4.4;10.1]	8.3 [5.5;11.6]	0.009
NLR, median (IQR)	12.6 [7.6;22.9]	11.3 [6.8;19.2]	16.0 [9.8;27.0]	0.005
Glycosylated hemoglobin count, median (IQR)	6.3 [5.7;7.5]	6.2 [5.7;7.4]	6.8 [5.8;7.7]	0.048
28-day status				
Death	83 (35.3%)	56 (33.3%)	27 (40.3%)	0.391
Still in ICU	11 (4.7%)	4 (2.4%)	7 (10.4%)	0.014
Still in hospital	17 (7.2%)	9 (5.4%)	8 (11.9%)	0.095
Discharged	124 (52.8%)	99 (58.9%)	25 (37.3%)	0.004

BMI, Body mass index; Normal: 18.5≤BMI<24; Underweight: BMI ≤ 18.5; Overweight:24≤BMI<28; Obesity: BMI≥28; COVID-19 severity: reference the diagnosis and treatment plan for novel coronavirus infection (10th edition, trial); APACHE II score: the Acute Physiology and Chronic Health Evaluation II score; SOFA score: Sequential Organ Failure Assessment; COPD: Chronic obstructive pulmonary disease. Onset to admission: When symptoms such as fever, cough, sore throat, difficulty breathing, etc. occur to the time of admission; IMV: Invasive mechanical ventilation; CRRT: Continuous renal replacement therapy; Serum GM positive: Serum galactomannan test≥0.25; Serum BDG positive: Serum (1,3)-β-D-glucan test≥70. NLR: Neutrophil Lymphocyte ratio.

### 3.1 Types of fungi

**Figure 2:**
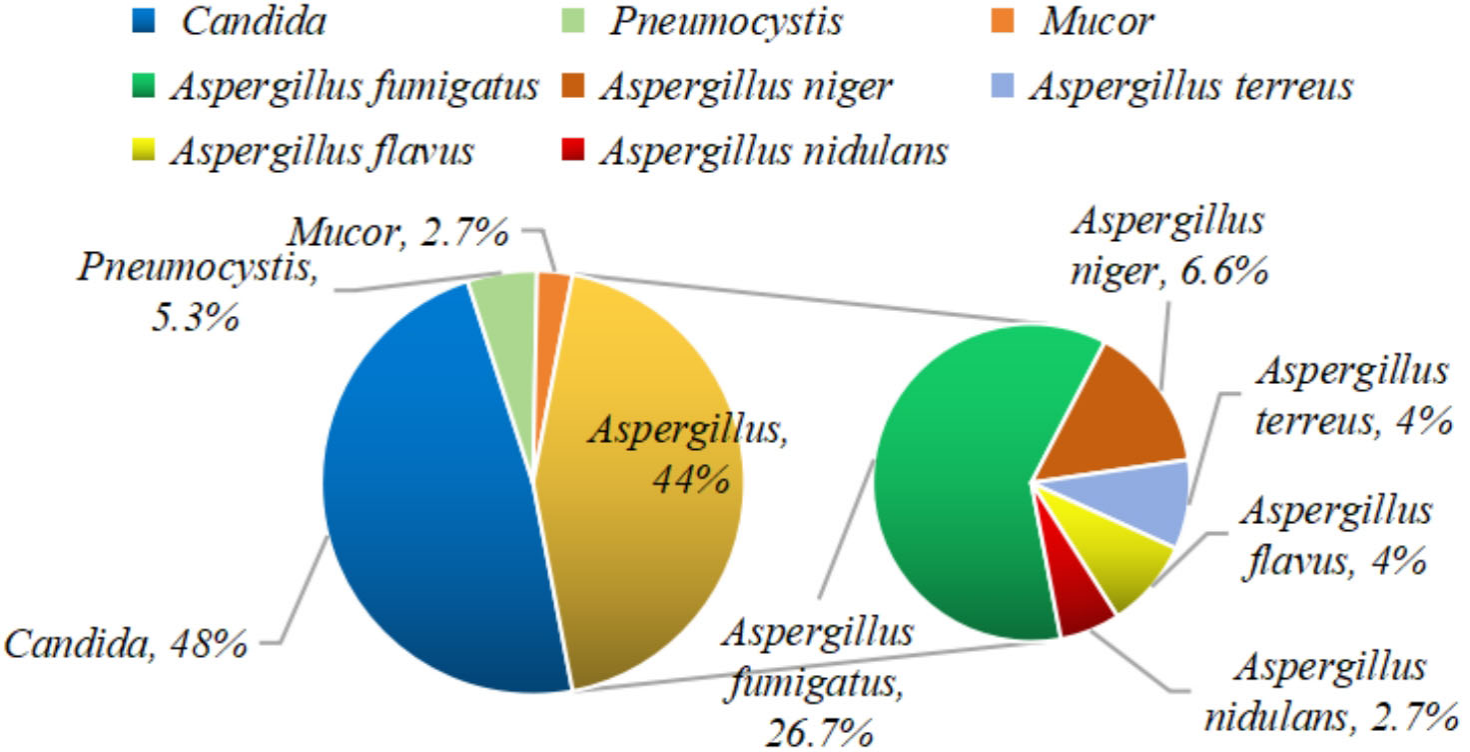
Fungal species proportion chart.

In the 67 patients with secondary IFI following COVID-19, a total of 75 fungal strains were detected, with 9 patients having co-infections involving at least two different fungal species. The most common fungal genus was Candida, accounting for 48% (36/75) of the cases, followed by Aspergillus, which constituted 44% (33/75). Pneumocystis accounted for 5.3% (4/75) of the cases, while Mucor was found in 2.7% (2/75) (**Figure 2**).

### 3.2 Univariate analysis of IFI

#### 3.2.1 Baseline characteristics before admission

The median time from onset of symptoms (fever, cough, sputum, dyspnea, etc.) to hospital admission for patients with secondary IFI of COVID-19 was 7 days (IQR 5.5-11.5 days). There were no statistically significant differences in underlying diseases (diabetes, cardiovascular, chronic lung diseases) between the IFI and non-IFI groups. However, it’s worth noting that a higher proportion of patients in the IFI group reported long-term glucocorticoid use before admission (16.4% vs. 7.1%, p=0.055).

#### 3.2.2 Laboratory indicators

A greater number of patients with IFI were classified as having a “critical” COVID type. Specifically, 21 out of 67 (31.3%) patients with IFI tested positive on the serum GM test, compared to 11 out of 168 (6.5%) in patients without IFI (p < 0.001). Additionally, 46.3% vs. 25% tested positive on the BDG-test respectively (p = 0.002). Upon admission, a significantly higher proportion of patients in the IFI group had Sequential Organ Failure Assessment (SOFA) scores greater than 2 (p = 0.012), and a positive influenza virus nucleic acid test (p = 0.081). Patients with IFI also displayed higher average white blood cell counts (9.8 [6.4, 12.9] vs. 7.6 [5.2, 11.4], p = 0.021), elevated neutrophil counts (8.3 [5.5, 11.6] vs. 6.3 [4.4, 10.1], p = 0.009), and an increased neutrophil-to-lymphocyte ratio (NLR) (16 [9.8, 27.0] vs. 11.3 [6.8, 19.2], p = 0.005), as compared to patients without IFI. Moreover, the log-transformed IL-6 level was also significantly higher in patients with IFI (p = 0.003).

#### 3.2.3 Treatment/intervention after admission

COVID-19 patients mostly received anticoagulant therapy (82.6%), and Nirmatrelvir/Ritonavir was the most commonly used antiviral drug (41.7%). Patients with IFI were more less to use Azivudine compared to non-IFI patients (16.4% vs. 32.1%, p=0.023). Regarding immunotherapy (corticosteroids, Tocilizumab), we did not find statistically significant data. It is noteworthy that the likelihood of COVID-19 patients developing IFI increased significantly with invasive procedures, including invasive mechanical ventilation (IMV) (58.2% vs. 32.7%, p=0.001), central venous catheterization (52.2% vs. 33.3%, p=0.011), fiberoptic bronchoscopy (34.3% vs. 11.9%, p<0.001), and tracheostomy (22.4% vs. 8.3%, p=0.006). IFI patients had a higher proportion of using broad-spectrum antibiotics for more than 2 weeks (62.7% vs. 26.8%, p<0.001) and more frequently used vasoactive drugs (50.7% vs. 32.7%, p=0.016).

#### 3.2.4 28-day status

Patients diagnosed with IFI had significantly longer stay in the ICU compared to patients without IFI (median 13 [9, 16.5] days vs. median 9 [5, 13] days, p < 0.001). After 28 days, there were a total of 11 COVID-19 patients (4.7%) still hospitalized in the ICU, with the majority belonging to the IFI group (10.4% vs. 2.4%,p=0.014), who had a lower likelihood of being discharged within 28 days (33.7% vs. 58.9%,p = 0.004). However, we did not observe a significant difference in mortality rates.

### 3.3 Univariate analysis of CAPA

Of the 67 patients, 30 (44.8%) had CAPA. Regarding underlying conditions, we found that patients with CAPA were more likely to have diabetes (66.7% vs. 24.3%, p=0.001), and all four patients with chronic bronchitis were from the CAPA group. Additionally, patients in the CAPA group exhibited a significantly higher rate of positive GM test results (66.7% vs. 2.7%, p<0.001), while the positive BDG test rate did not show strong statistical significance in the CAPA group. Furthermore, no other statistically significant differences in characteristics were observed between the CAPA and non-CAPA groups (**Table 2**).

**Table 2:** Characteristics and related clinical factors by COVID-19-associated pulmonary aspergillosis (CAPA) and other fungi (non-CAPA) in all IFI cases.

Characteristics	All IFI cases	non-CAPA	CAPA	P-value
	N=67	N=37	N=30	
Age(years), median (IQR)	71.0 [66.0;78.0]	72.0 [60.0;78.0]	71.0 [68.0;75.8]	0.791
BMI (kg/m^2^), median (IQR)	23.2 [21.3;25.4]	23.6 [21.0;25.6]	23.0 [21.7;25.2]	0.777
ICU stay, median (IQR)	13.0 [9.0;16.5]	13.0 [8.0;16.0]	12.5 [10.0;17.0]	0.487
Onset to admission, median (IQR)	7.0 [5.5;11.5]	7.0 [4.0;10.0]	7.5 [7.0;14.5]	0.269
Severity scores				
Type I respiratory failure	56 (83.6%)	30 (81.1%)	26 (86.7%)	0.742
Type II respiratory failure	10 (14.9%)	5 (13.5%)	5 (16.7%)	0.743
Lymphopenia	54 (80.6%)	29 (78.4%)	25 (83.3%)	0.842
Neutropenia	1 (1.5%)	0 (0.0%)	1 (3.3%)	0.448
Basic illness				
Diabetes	29 (43.3%)	9 (24.3%)	20 (66.7%)	0.001
Hematologic malignancies	1 (1.5%)	0 (0.0%)	1 (3.3%)	0.448
COPD	6 (9.0%)	1 (2.7%)	5 (16.7%)	0.082
Chronic bonchitis	4 (6.0%)	0 (0.0%)	4 (13.3%)	0.036
Laboratory data				
Serum GM positive	21 (31.3%)	1 (2.7%)	20 (66.7%)	<0.001
Serum BDG positive	31 (46.3%)	14 (37.8%)	17 (56.7%)	0.197
Log ^IL-6^, median (IQR)	3.2 [1.7;4.6]	2.8 [1.2;4.3]	3.7 [2.6;4.6]	0.072
Log ^CRP^, median (IQR)	4.4 [3.4;5.0]	4.4 [3.4;5.0]	4.4 [3.4;4.9]	0.588
Glycosylated hemoglobin count, median (IQR)	6.8 [5.8;7.7]	5.9 [5.6;7.4]	7.3 [6.7;8.3]	<0.001
Antiviral treatment				
Nematrevir/ritonavir	31 (46.3%)	16 (43.2%)	15 (50.0%)	0.76
Azivudine	11 (16.4%)	7 (18.9%)	4 (13.3%)	0.742
Monolaurin	3 (4.5%)	0 (0.0%)	3 (10.0%)	0.085
Anticoagulant therapy	56 (83.6%)	28 (75.7%)	28 (93.3%)	0.095

Type I respiratory failure: PaO_2_<60mmHg; Type II respiratory failure: PaO_2_<60mmHg and PaCO_2_>50mmHg; Lymphopenia: Lymphocyte count absolute value<0.8*10^9^; Neutropenia: Absolute neutrophil count<1.5*10^9^.

### 3.4 Multivariate analysis of IFI

Using stepwise model selection, we inputted 44 variables in the model selection. The resulting model included 7 factors, including the use of broad-spectrum antibiotics for more than 2 weeks (aOR=4.14, 95% CI 2.03-8.67), high BMI (aOR=0.85, 95% CI 0.75-0.94), fever (aOR=2.3, 95% CI 1.16-4.55), elevated log ^IL-6^ levels (aOR=1.22, 95% CI 1.04-1.43), prone position ventilation (aOR=2.38, 95% CI 1.15-4.97),the use of COVID-19 immunoglobulin (aOR=0.45, 95% CI 0.2-0.97) and positive influenza virus nucleic acid test (aOR=3.35, 95%CI 0.78-14.65). (**Table 3**). Due to the limited sample size of our CAPA data, there is a relatively larger margin of error in the multivariate analysis. Therefore, we present the multivariate analysis of CAPA as **Supplementary Material**, for reference purposes only.

**Table 3:** Multivariable logistic regression results for prediction of IFI in COVID-19 patients.

Variables	coefficient	aOR	95% CI	P-value
**Broad-spectrum antibiotics greater than 2 weeks**	**1.42**	**4.14**	**2.03-8.67**	**<0.001**
**High BMI**	**-0.17**	**0.85**	**0.75-0.94**	**0.003**
**Fever**	**0.83**	**2.3**	**1.16-4.55**	**0.017**
**Log ^IL-6^**	**0.19**	**1.22**	**1.04-1.43**	**0.016**
**Prone position ventilation**	**0.87**	**2.38**	**1.15-4.97**	**0.02**
**COVID-19 immunoglobulin**	**-0.8**	**0.45**	**0.2-0.97**	**0.047**
Positive influenza virus nucleic acid	1.21	3.35	0.78-14.65	0.099

These are the model covariates we selected; bold text is the significant results; aOR, adjusted Odds Ratio; CI, confidence intervals.

### 3.5 Evaluation of the logistic regression prediction model

We conducted statistical analysis using the logistic regression prediction model to calculate the predicted probability (P) of individual IFI occurrence. We generated ROC curves based on the predicted values and true values. The AUC for the model derived from **Table 3** was 0.81. An AUC value between 0.8-0.9 is considered good, indicating that both models have meaningful predictive capabilities.

## 4 Discussion

In this retrospective study, we have reported on the cumulative incidence and mortality of secondary IFI and CAPA in severe COVID-19 patients in the ICU and established a logistic regression prediction model through multivariate analysis. Prior to the emergence of the COVID-19 pandemic, the incidence of IFI in the ICU was relatively low, with only 18.9 cases per 1000 admissions (Montagna et al., 2013). However, with the advent of SARS-COV-2 infection, the incidence of IFI has surged nearly 15-fold, with invasive candidiasis and CAPA emerging as the most prevalent forms. Previous studies have reported that the incidence of secondary IFI due to COVID-19 in observational studies conducted in ICU settings ranged from 2.5% to 33.3% (Chong et al., 2021). The incidence observed in our study, at 28.5%, falls on the higher end of this spectrum. This wide variation in incidence may be attributed to differences in the classification criteria used for fungal infections, challenges in early-stage diagnosis, variations in the waves of SARS-COV-2 variants, differences in study monitoring protocols, hospital environment management, and variation in the patient population studied.

An observational study (Casalini et al., 2021) has indicated that meaningful comparisons of fungal infection incidence rates are challenging when different classification criteria are employed. Such comparisons are greatly influenced by variations in the study environment, whether it be in an ICU or a general ward. The ICU setting is advantageous for obtaining lower respiratory tract samples, thus bolstering fungal detection during screening. Conversely, in general wards, reliance on tests like serum BDG and GM is constrained for diagnosing this disease. This study was conducted within an ICU environment, which can account for the elevated incidence rate of IFI. This finding aligns with the heightened susceptibility of critically ill ICU patients to IFI, as documented in previous studies (Montagna et al., 2013; CDC, 2022).

Among COVID-19 patients in the ICU, the overall incidence of CAPA ranged from 1% to 39.1% (Salmanton-García et al., 2021), making it the most common IFI in COVID-19 patients. In this study, the incidence of CAPA was 12.8%, which falls towards the lower end of this range. The reduced incidence can be attributed to a lack of screening, effective testing, and the availability of fiberoptic bronchoscopy. Our study defined CAPA using the newly proposed ECMM/ISHAM criteria (Koehler et al., 2021), which has demonstrated significant benefits in CAPA management, bringing CAPA incidence closer to autopsy studies (Prattes et al., 2021; Gangneux et al., 2022). Although biomarkers for detecting IFI such as serum GM and BDG, or upper respiratory secretions, are more convenient for clinical collection, they have limited utility in detecting fungal DNA at early phase of infection (Alanio et al., 2020; Nasir et al., 2020; Roman-Montes et al., 2021).Therefore, our detection methods for fungi rely more on bronchoscopy to collect BAL for mNGS, and this invasive procedure is associated with aerosol generation, increasing the risk of infection for healthcare personnel. Despite using protective equipment to enhance bronchoscopy availability, factors such as severe respiratory distress syndrome (ARDS) and hemodynamic instability may render the procedure challenging (Shadrivova et al., 2021). In our study population, the majority of critically ill patients requiring vasopressor therapy were unable to tolerate bronchoscopy, accounting for 81.7% (192/235) of invasive procedures. As a result, the collected BAL sample volume is relatively low. Additionally, mNGS has a relatively high cost, and some patients or their families may be unable to afford the cost of this fungal detection, potentially leading to a further decrease in the rate of fungal detection.

In terms of mortality, among patients with IFI in our study, the 28-day mortality rate was 40.3%. While no significant difference in mortality was observed between patients with and without IFI, those with IFI had longer ICU stays (median 13 days [IQR 9-16.5 days]), prolonged mechanical ventilation (mean 4.6 days [IQR 0-10.8 days]), worsening expiratory support, and a greater susceptibility to hemodynamic instability. One possible reason for the lack of significant mortality difference could be the time limitation, as we did not record the survival information of some COVID-19 patients who were hospitalized for more than 28 days. Although there was a trend toward higher 28-day mortality among patients in the IFI group, it did not reach statistical significance when compared with the non-IFI group.

ICU patients are at an increased risk of undergoing invasive procedures such as central venous catheter insertion or tracheotomy. A retrospective study of opportunistic candida infections in COVID-19 patients in Turkey demonstrated that more than 70% of patients with central venous catheters developed Central Venous Catheters (CVCs)-associated infections (Altinkaya Çavuş and Sav, 2022). In our study, 93.2% of COVID-19 patients were classified as severe or critical, and the severity of COVID-19 is closely linked to the development of IFI, as reported in the MYCOVID study (Gangneux et al., 2022) and the Leistner et al. study (Leistner et al., 2022). Consequently, this population is at extremely high risk for secondary IFI during their hospitalization.

Research indicates that prolonged use of broad-spectrum antibiotics such as meropenem or moxifloxacin in severe COVID-19 patients, while effective against a broad range of bacteria, may increase Candida colonization. This can disrupt the interaction between bacterial and fungal microbiomes, leading to increased pathogenicity of Candida (Laudenbach and Epstein, 2009). Our study supports this finding, as multivariate analysis revealed that patients who used broad-spectrum antibiotics for more than 2 weeks during ICU hospitalization had a 4.14-fold increased odds of developing IFI.

In the early stages of the SARS-CoV-2 outbreak in Wuhan in 2019, a study in China showed a 100% antibiotic usage rate in severe COVID-19 patients (Du et al., 2020). Upon hospital admission of COVID-19 patients, if there was a significant increase in white blood cell and neutrophil counts, empirical broad-spectrum antimicrobial treatment was initiated due to suspicion of community-acquired pneumonia or secondary bacterial infection. Microbial screening was conducted simultaneously, and once bacterial infection was confirmed, targeted therapy was promptly initiated. However, research suggests that the use of broad-spectrum antibiotics, whether for empirical or targeted treatment of dual infections in COVID-19 patients, increases the likelihood of fungal infections caused by endogenous fungi (Laudenbach and Epstein, 2009; Du et al., 2020). In our study, the antibiotic treatment duration for COVID-19 patients was mostly 7-10 days. Nevertheless, some patients experienced a subsequent rise in inflammatory indicators such as white blood cells or neutrophils shortly after discontinuation of antibiotics. Consequently, the use of broad-spectrum antibiotics had to be extended. Given the results from our prediction model, we advocate minimizing the duration of antibiotic use to reduce the likelihood of dual bacterial and fungal infections.

The majority of CAPA cases occurred in COVID-19 patients with underlying diseases such as long-term (median 15 days) lymphopenia, decompensated diabetes, and hematologic malignancies (Rutsaert et al., 2020). In univariate analysis, our study found that diabetes, as an underlying condition, had statistical significance in CAPA patients. This suggests that elevated blood glucose levels in the body may contribute to the formation and pathogenicity of Aspergillus in these patients.

Furthermore, our prediction model, indicates that patients with elevated log ^IL-6^ levels at admission and those experiencing fever were independently associated with 1.22 and 2.3 times the odds of developing IFI, respectively. It is widely acknowledged that severe COVID-19 is characterized by immune dysregulation, with IL-6 playing a central role as a key regulator of adaptive immunity. The progression from initial SARS-CoV-2 infection to a more complex disease is driven by an excessive host immune response and autoimmune damage. Overactivation of IL-6 is a pivotal mediator in the development of COVID-19 into respiratory failure, shock, and multiple organ dysfunction. Studies have indicated that patients with severe COVID-19 exhibit elevated levels of pro-inflammatory cytokines (IL-6, tumor necrosis factor-α) and anti-inflammatory cytokines (IL-4, IL-10), along with decreased expression of CD4 and CD8 cells (Chen et al., 2020; Tay et al., 2020). This intricate immune landscape heightens the risk of IFI, leading to lung tissue damage and mucosal destruction, creating an environment conducive to fungal infection and fostering the proliferation of lung microorganisms (Cunha et al., 2012; Tolle and Standiford, 2013; Arastehfar et al., 2020). Nevertheless, the independent relationship between IFI secondary to SARS-CoV-2 infection and high IL-6 levels has not been consistently demonstrated in previous studies. Whether IL-6 plays a role in modulating innate immunity and adaptability during fungal infection, thus contributing to the body’s susceptibility to IFI, necessitates further research for confirmation.

From the perspective of treating COVID-19 patients, immunotherapy is the primary choice for the treatment of most COVID-19 patients, as the anti-inflammatory properties of immunosuppressive agents are crucial in counteracting the increased release of pro-inflammatory cytokines in the lungs during SARS-CoV-2 infection, a phenomenon referred to as the “cytokine storm.” (Ye et al., 2020). Our prediction model also includes variables related to immunotherapy for COVID-19 patients, specifically the use of methyl prednisolone. Although it is a non-significant predictor variable in our model, studies have confirmed that the application of corticosteroids significantly increases the odds of developing IFI (White et al., 2021). Similarly, Riche et al. (Riche et al., 2020) observed a ten-fold increase in the prevalence of Candida among severe COVID-19 patients receiving high-dose corticosteroids. However, it’s important to note that some studies have raised concerns about the long-term administration of high-dose corticosteroids for treating SARS-Cov-2 infection due to the high incidence of systemic fungal infections. A large retrospective study conducted in New York advocated for the early use of low-dose corticosteroids in COVID-19 treatment, finding no association between corticosteroid use and increased fungal infections (Ho et al., 2021). Although we advocate for low-dose, short-term corticosteroid treatment in COVID-19 patients, for those with extensive pulmonary inflammation and infiltration, we administer short-term, high-dose methyl prednisolone pulse therapy. This, however, inevitably leads to immune imbalance, significantly increasing the chances of opportunistic pathogen infections such as Aspergillus and Candida. Therefore, we recommend conducting targeted research on such immunosuppressive agents, with the hope of establishing a specific dosage range. This would aim to optimize the treatment of COVID-19 patients without simultaneously increasing the infection rate of IFI.

The use of COVID-19 immunoglobulin is a significant variable in our prediction model. Immunoglobulin, also known as antibodies, is a protein produced by the body’s immune system. In our treatment of COVID-19 patients with COVID-19 immunoglobulin, the primary goal is to provide short-term passive immune support. By introducing these antibodies into the patient’s body, we can enhance the patient’s immune response and help combat the virus. However, a large clinical trial focused on immunoglobulin therapy for COVID-19 patients found no clinical benefit for COVID-19 patients after the administration of immunoglobulin (7 days post-infusion). They suggested that antibody treatment might not be beneficial for patients who have already mounted an immune response (Group, 2022). Furthermore, some scholars believe that in patients who have progressed to the stage of COVID-19 pneumonia, the infusion of antibodies may not effectively penetrate lung tissues. Therefore, enhancing humoral immune response might not be effective in such cases (Griffin et al., 2021). In our use of immunoglobulin therapy for COVID-19 patients, it is applied to severe or rapidly progressing cases, following the principles of early intervention and short duration (not exceeding a total of 5 doses). This approach may be beneficial as we strictly limit the timeframe. Additionally, our prediction model indicates an adjusted odds ratio (aOR) of 0.45, with a 95% confidence interval of 0.2-0.97. This suggests that early administration of immunoglobulin in the course of the disease may protect the body from fungal infections, largely due to the immunomodulatory effects enhancing the patient’s immune response. Considering no other study has reported the effect of immunoglobulin use on IFI risks, we believe further research and observation are needed.

It is worth noting that our prediction model also includes a protective variable – patients with a high BMI (obesity) are less susceptible to IFI. The correlation between obesity and IFI secondary to COVID-19 has been scarcely reported, and published data have indicated that high BMI is negatively associated with protective immune responses. BMI over 30 kg/m^2^ is considered a risk factor for fungal infection (Falagas and Kompoti, 2006; Karlsson and Beck, 2010). During the 2009 influenza A(H1N1) pandemic, several studies unequivocally demonstrated that obese and morbidly obese individuals were more susceptible to infection with the influenza A(H1N1) pdm09 virus and experienced more severe illness upon infection (Jain et al., 2009; Vaillant et al., 2009). Regarding obesity, Frasca et al. proposed a concept referred to as “obesity paradox” (Frasca and McElhaney, 2019). In their study, they compared the outcomes of influenza and pneumococcal pneumonia, discovering that advanced age and obesity could both lead to severe complications from influenza. However, with increasing age, obesity appears to have a protective effect against severe complications from pneumococcal pneumonia. Our research findings can also provide an explanation for this paradox. In our analysis among ICU patients with a median age of 72, we found that being in the obese BMI group (BMI ≥28 kg/m^2^) was associated with 0.85 (95% CI 0.75-0.94) times the odds of developing IFI compared to the normal BMI group (BMI between 18.5-23.9 kg/m^2^). A clear explanation is that, particularly in severe patients, especially the elderly, higher body weight may signify a better energy reserve, which can protect against certain infections. Moreover, immune responses are altered in obese individuals, and well-nourished obese patients may have more resources to support their immune function during an infection. Additionally, we used a Chinese-specific obese cutoff of 28 in this study, which may explain the contradictory findings compared to other studies. Future research is urgently needed to address significant gaps in understanding the complex interplay between obesity and infection in vulnerable populations.

A major strength of this study lies in its provision of detailed and comprehensive information on the incidence, clinical characteristics, and outcomes of IFI in COVID-19 patients in Chinese ICUs. By establishing a prediction model for secondary IFI in COVID-19 patients, we also offer initial insights into various potential risk factors, contributing to a better understanding of the mechanisms behind IFI and the development of improved prevention and treatment strategies. However, we are acutely aware of the limitations of our research, which is a retrospective, single-center cohort study. We did not conduct virus molecular typing studies, so we cannot determine if these infections are associated with specific pathogen strains. Our fungal infection detection is primarily conducted using respiratory samples collected through bronchoscopy, which limits the number of samples we can gather. We also relied on clinical decisions by physicians to perform bronchoscopy, which may introduce selection bias. Furthermore, due to the limited sample size of CAPA in our study, we faced challenges in conducting a robust multivariate analysis, and there is a relatively larger margin of error.

## 5 Conclusion

In summary, during the first wave of the COVID-19 pandemic following the relaxation of lockdown measures, we observed a cumulative incidence of 28.5% (67/235) for secondary invasive fungal infections in severe COVID-19 patients in the ICU, with COVID-19-associated pulmonary aspergillosis constituting a cumulative incidence of 12.8% (30/235). Integrating variables from our prediction model for COVID-19-associated secondary invasive fungal infections, we recommend paying special attention to patients with low BMI (BMI < 28 kg/m^2^), elevated log ^IL-6^ levels and fever in severe COVID-19 patients. This is because patients with these characteristics are more susceptible to IFI. Furthermore, in the treatment of COVID-19 patients, we emphasize the importance of minimizing the duration of broad-spectrum antibiotic use, and the use of COVID-19 immunoglobulin may also reduce the incidence of IFI.

## Data availability statement

The original contributions presented in the study are included in the article/**Supplementary Material**. Further inquiries can be directed to the corresponding author.

## Ethics statement

The studies involving humans were approved by The research protocol has been approved by the Ethics Committee of China-Japan Union Hospital, Jilin University. (Approval No: 2023-KYYS-092). The studies were conducted in accordance with the local legislation and institutional requirements. The participants provided their written informed consent to participate in this study. Written informed consent was obtained from the individual(s) for the publication of any potentially identifiable images or data included in this article.

## Author contributions

LS: Conceptualization, Data curation, Investigation, Methodology, Writing – original draft. TY: Data curation, Formal Analysis, Writing – review & editing. CZ: Supervision, Writing – review & editing. PH: Supervision, Writing – review & editing. ZZ: Methodology, Supervision, Validation, Writing – review & editing.
