# Supplementary material for: A prediction model for secondary invasive fungal infection among severe SARS-CoV-2 positive patients in ICU
Source: Front Cell Infect Microbiol. 2024 Jul 8;14:1382720. doi: 10.3389/fcimb.2024.1382720 (PMC11260608; doi:10.3389/fcimb.2024.1382720)
Supplement: Supplementary file 1 [file DataSheet_1.pdf]

Supplementary Material

1 Supplementary Tables

1.1 Multivariable logistic regression results for prediction of CAPA in COVID-19 patients.

Variables	coefficient	aOR	95% CI	P-value
**High glycosylated hemoglobin**	**1.75**	**5.75**	**2.38-21.17**	**0.001**
**Elevated log $^{IL-6}$**	**0.56**	**1.75**	**1.04-3.33**	**0.05**
**Methyl prednisolone**	**2.22**	**9.2**	**1.62-82.88**	**0.024**
**Tracheotomy**	**-3.17**	**0.04**	**0-0.37**	**0.01**
**Prone position ventilation**	**3.52**	**33.66**	**3.46-662.84**	**0.007**
Broad-spectrum antibiotics greater than 2 weeks	1.52	4.56	0.89-31.93	0.087
**Fever**	**-2.5**	**0.08**	**0.01-0.48**	**0.012**
**COVID-19 immunoglobulin**	**-3.03**	**0.05**	**0-0.5**	**0.019**
Deterioration ventilatory parameters	-2.05	0.13	0.01-0.9	0.059
TNF- α	0.56	1.75	0.93-4.77	0.2
Hypertension	-1.42	0.24	0.03-1.45	0.149

bold text is the significant results.
